# SIN3A and SIN3B differentially regulate breast cancer metastasis

**DOI:** 10.18632/oncotarget.12805

**Published:** 2016-10-21

**Authors:** Monica J. Lewis, Jianzhong Liu, Emily Falk Libby, Minnkyong Lee, Nigel P.S. Crawford, Douglas R. Hurst

**Affiliations:** ^1^ Department of Pathology, University of Alabama at Birmingham, Birmingham, AL, USA; ^2^ Genetics and Molecular Biology Branch, National Human Genome Research Institute, National Institutes of Health, Bethesda, MD, USA

**Keywords:** SIN3A, SIN3B, breast cancer, invasion, metastasis

## Abstract

SIN3 corepressor complexes play important roles in both normal development and breast cancer. Mammalian cells have two paralogs of SIN3 (SIN3A and SIN3B) that are encoded by distinct genes and have unique functions in many developmental processes. However, specific roles for SIN3A and SIN3B in breast cancer progression have not been characterized. We generated stable knockdown cells of SIN3 paralogs individually and in combination using three non-overlapping shRNA. Stable knockdown of SIN3B caused a significant decrease in transwell invasion through Matrigel and decreased the number of invasive colonies when grown in a 3D extracellular matrix. Conversely, stable knockdown of SIN3A significantly increased transwell invasion and increased the number of invasive colonies. These results were corroborated *in vivo* in which SIN3B knockdown significantly decreased and SIN3A knockdown increased experimental lung metastases. RNA sequencing was used to identify unique targets and biological pathways that were altered upon knockdown of SIN3A compared to SIN3B. Additionally, we analyzed microarray data sets to identify correlations of SIN3A and SIN3B expression with survival in patients with breast cancer. These data sets indicated that high mRNA expression of SIN3A as well as low mRNA expression of SIN3B correlates with longer relapse free survival specifically in patients with triple negative breast cancer which corresponds with our *in vitro* and *in vivo* data. These results demonstrate key functional differences between SIN3 paralogs in regulating the process of breast cancer metastasis and suggest metastasis suppressive roles of SIN3A and metastasis promoting roles of SIN3B.

## INTRODUCTION

The stage-specific five-year relative survival rate for patients with distant breast cancer metastases at diagnosis is approximately 25%; a rate that has not significantly changed in the past two decades and is compared to a relative five-year survival rate of near 100% for patients with localized disease [1]. Patients with metastatic breast cancer have limited treatment options, signifying the need for more studies to better understand metastasis at the molecular level. The process of metastasis is highly complex and inefficient. Tumor cells that have the potential to metastasize must respond to different microenvironments for continued survival and proliferation by expressing specific gene sets [2, 3]. It has become increasingly clear that many of these metastasis-associated genes are regulated epigenetically by chromatin remodeling complexes that play important roles in normal development [4–6].

A key epigenetic regulator that is required for normal development and has been implicated in breast cancer progression is SIN3 (Switch-Independent 3) [7, 8]. SIN3 is a scaffolding protein that regulates gene transcription through chromatin modification by recruiting histone deacetylases (HDACs) to function at particular sites of the genome, typically leading to transcriptional silencing although several genes have been shown to be activated [9–11]. Mammalian cells have two SIN3 paralogs (SIN3A and SIN3B) that are encoded by genes on chromosomes 15 and 19 respectively. The proteins are 52% identical and 68% similar with regard to amino acid sequence and mouse Sin3A is more similar to human SIN3A than to mouse Sin3B. Knockout of either paralog is lethal, but at different stages of development and several unique defects were identified in the *Sin3A* compared to *Sin3B* knockout [12–15]. However, mechanisms for paralog specific functions during embryonic development are yet to be fully elucidated.

Recent studies have suggested regulatory roles for SIN3 complexes in breast cancer progression. Inhibition of protein-protein interaction within the second paired amphipathic helix (PAH2) region of SIN3 with a SIN3-interacting domain decoy peptide resulted in induction of differentiation of metastatic breast cancer cells and inhibition of breast cancer cell invasion and metastasis [16, 17]. Following *in silico* screening for small molecules that interact with SIN3, macrocyclic lactone derivatives of avermectin were identified that were effective at reducing invasion and metastasis of triple negative breast cancer cell lines [18]. Additionally, significant suppression of breast cancer metastasis was demonstrated with alterations to the composition of SIN3 protein complexes [19]. These studies suggest SIN3 complexes are promoters of tumor progression. However, in each of those studies, specific functions for SIN3A or SIN3B were not determined. Das *et al*. showed that SIN3A inhibits invasion using a *Drosophila* model and RNAi-mediated knockdown of dSin3 led to increases in cell migration, invasion and epithelial-to-mesenchymal transition [20]. In pancreatic cancer, Sin3B was found to promote cancer progression by senescence-associated inflammation [21]. Although these studies provide clear evidence for the involvement of SIN3 complexes in cancer, it is not understood how or under what context SIN3 complexes favor suppressive or promoting functions for tumor progression.

We hypothesized that SIN3A and SIN3B play differential roles during breast cancer progression. Here, we show that individual knockdown of SIN3A causes an increase, whereas knockdown of SIN3B causes a decrease in breast cancer invasion and metastatic potential. Dual knockdown of both SIN3A and SIN3B mimics the individual knockdown of SIN3B. Unique targets and biological pathways were identified for SIN3A and SIN3B. Expression of *SIN3A* and *SIN3B* in patient data sets correlates with the *in vitro* and *in vivo* experimental data. The results suggest that SIN3B is required for and is a promoter of breast cancer progression and metastasis, and further suggest that SIN3A is a metastasis suppressor.

## RESULTS

### SIN3A and SIN3B regulate metastatic potential of breast cancer cells differently

**Figure 1:**
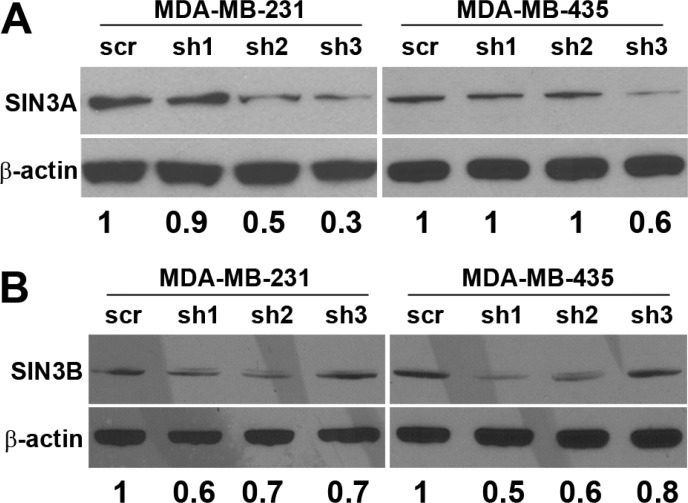
Knockdown of SIN3A and SIN3B Three non-overlapping shRNA constructs targeting sequences located in exon 19, 11, and 15–16 of *SIN3A* (sh1, sh2 and sh3 respectively) and exon 4, 20, and 3 of *SIN3B* (sh1, sh2, and sh3 respectively) (see Supplementary Table S1 for specific sequences) were stably transduced into MDA-MB-231 and −435. Whole cell lysates were probed by Western blot. Densitometry is shown under the blots as a ratio of SIN3A or SIN3B to β-actin normalized to the scrambled control (scr).

**Figure 2:**
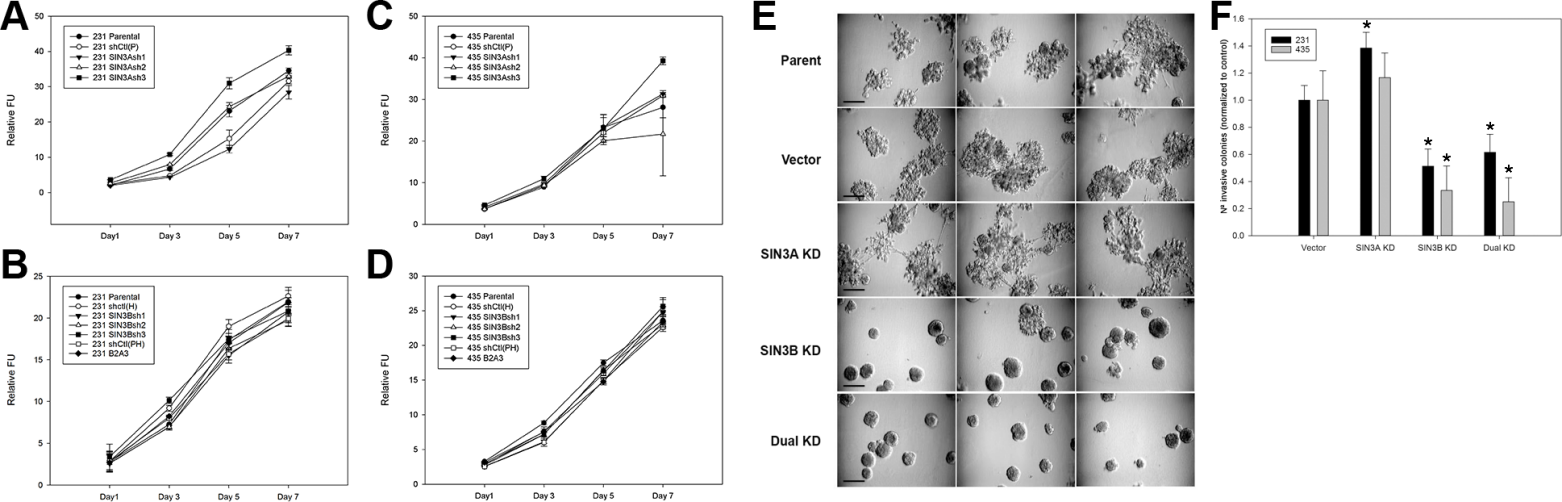
Morphological differences following knockdown of SIN3 paralogs in metastatic breast cancer cells (**A**, **B**) MDA-MB-231 and (**C**, **D**) MDA-MB-435 cells stably transduced with *SIN3A* or *SIN3B* targeting shRNA (see Supplementary Table S1 for sequences) were seeded on 96-well plates. Proliferation was assessed at times indicated using Alamar Blue reagent. No statistically significant differences were noted in the doubling times. (**E**) Cells were seeded on 24-well plates coated with Matrigel and incubated at 37°C for 10 days. Three representative images for MDA-MB-231 cells are shown. The sh3 construct was used for SIN3A and sh2 for SIN3B. Dual knockdown used both sh3 for SIN3A and sh2 for SIN3B as described in the Materials and Methods. A more invasive phenotype was noted for the SIN3A knockdown and epithelial-like phenotype noted for the SIN3B knockdown. Scale bar is 50 μm. (**F**) The number of invasive colonies (defined by colonies with spiculations) for each cell line was quantified and normalized to the vector control. *indicates *p* < 0.05 compared to vector control.

**Figure 3:**
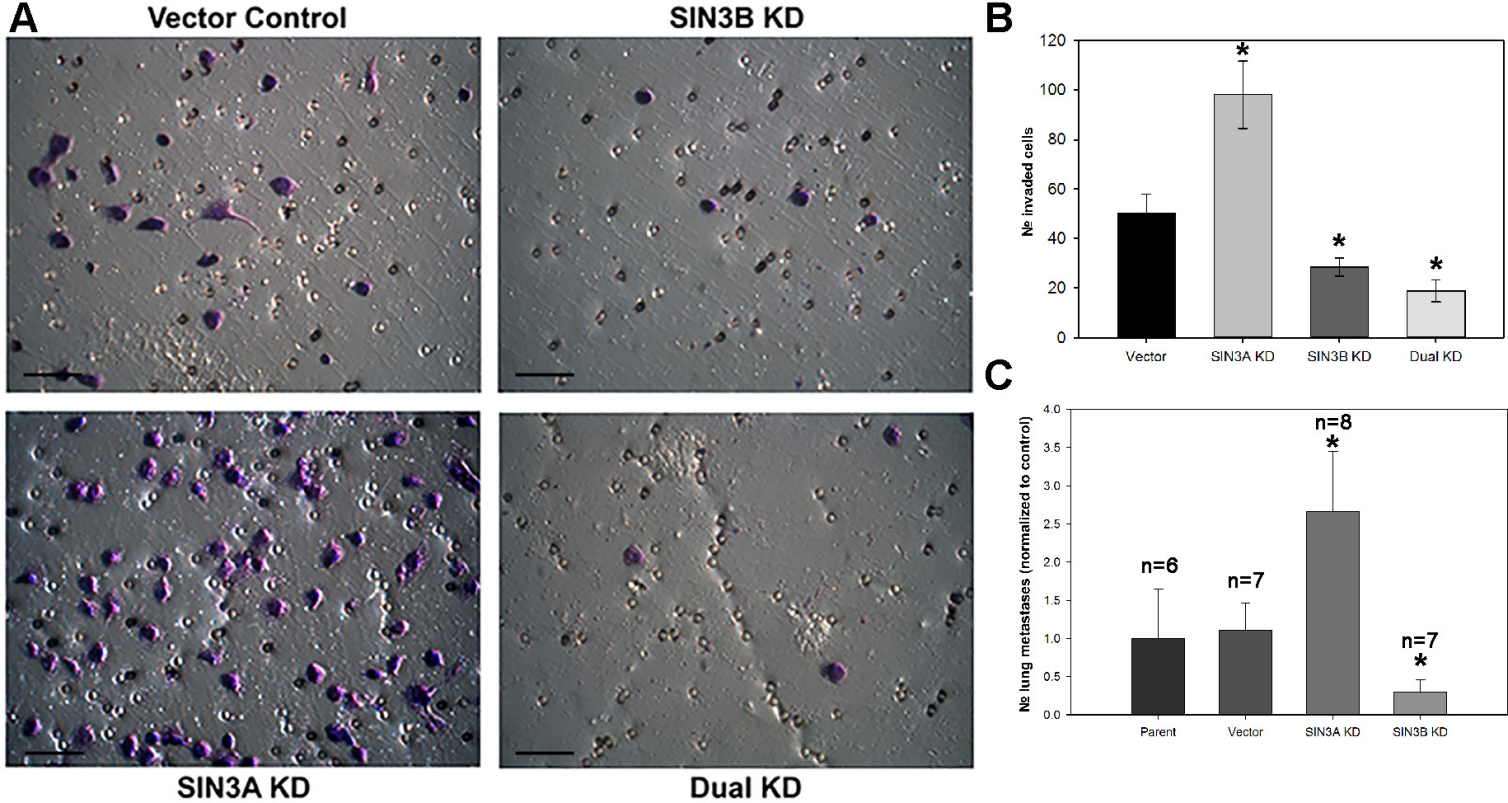
SIN3 paralogs inversely regulate invasion and metastasis (**A**) MDA-MB-231 cells were seeded into the inserts of 24-well plates coated with Matrigel in serum-free media. Photomicrographs were taken after 22-hour incubation period and staining with 1% crystal violet. The same shRNA constructs shown in Figure 2E were utilized. Scale bar is 50 μm. (**B**) The number of invaded cells was quantified. (**C**) MDA-MB-231 cells were injected into athymic mice *via* lateral tail vein. Lungs were collected 8 weeks post injection and stained in Bouin's fixative. Metastases per lung were counted and the average number was normalized to the parent control. Number of mice (n) is listed above each group. *indicates *p* < 0.05 compared to control.

To test whether SIN3 expression is required for breast cancer metastasis, we generated cell lines with stable knockdown of the SIN3 paralogs individually and in combination. We assessed 3 different non-overlapping shRNA targeting the individual paralogs (Supplementary Table S1). Because complete knockout of either SIN3 paralog is lethal, we utilized the shRNA constructs that resulted in approximately 50% knockdown (levels ranged between 30–70% Figure 1 and Supplementary Figure S1). Knockdown of SIN3A or SIN3B individually or in combination did not significantly affect proliferation (Figure 2A–2D). Likewise, no visible differences were noted in cell morphology when cells were grown in 2D tissue culture plates (Supplementary Figure S2). However, we observed striking differences in cell morphology when cells were grown in a 3D extracellular matrix (Figure 2E). Colonies with SIN3A knockdown were more invasive compared to the rounded epithelial-like colonies with SIN3B knockdown. We quantified the number of invasive versus non-invasive colonies compared to the vector control in MDA-MB-231 cells: SIN3A knockdown resulted in a 38% increase and SIN3B knockdown resulted in a 49% decrease of invasive colonies (Figure 2F). Dual knockdown of SIN3A and SIN3B was similar to SIN3B individual knockdown with a 38% decrease in invasive colonies. This is consistent with recent studies demonstrating inhibition of breast cancer invasion using SIN3 interacting domain decoy peptides and small molecule inhibitors of SIN3 that inhibit both SIN3A and SIN3B [16, 17]. The experiments were repeated in the MDA-MB-435 cell line with similar results: 17% increase of invasive colonies with SIN3A knockdown, 67% decrease with SIN3B knockdown, and 75% decrease with dual knockdown (Figure 2F). In support of these results, MDA-MB-436 followed the same trend with a 10% increase of invasive colonies for SIN3A knockdown and 17% decrease for SIN3B knockdown (data not shown). Cell invasion was further quantified with transwell invasion assays (Figure 3A, 3B). Again, we found an inverse relationship between SIN3 paralogs. Stable knockdown of SIN3A significantly increased invasion (95%, *p* = 0.008), whereas knockdown of SIN3B significantly decreased invasion (43%, *p* = 0.027). Dual knockdown of SIN3A and SIN3B decreased invasion similar to SIN3B knockdown (62%, *p* = 0.003). Together, these results demonstrate differential regulation of breast cancer cell invasion by SIN3A and SIN3B.

The significant differences in breast cancer invasion prompted us to characterize metastatic potential *in vivo* using an experimental metastasis model. Cells were injected into the lateral tail vein of athymic mice and the number of lung metastases was quantified. SIN3B knockdown decreased (70%) and SIN3A knockdown increased (160%) lung metastasis (Figure 3C) that correlates with our data from the *in vitro* invasion assays. These results further confirm the differential regulation of metastatic potential by SIN3A and SIN3B.

### Regulation of gene expression differs between SIN3A and SIN3B

**Figure 4:**
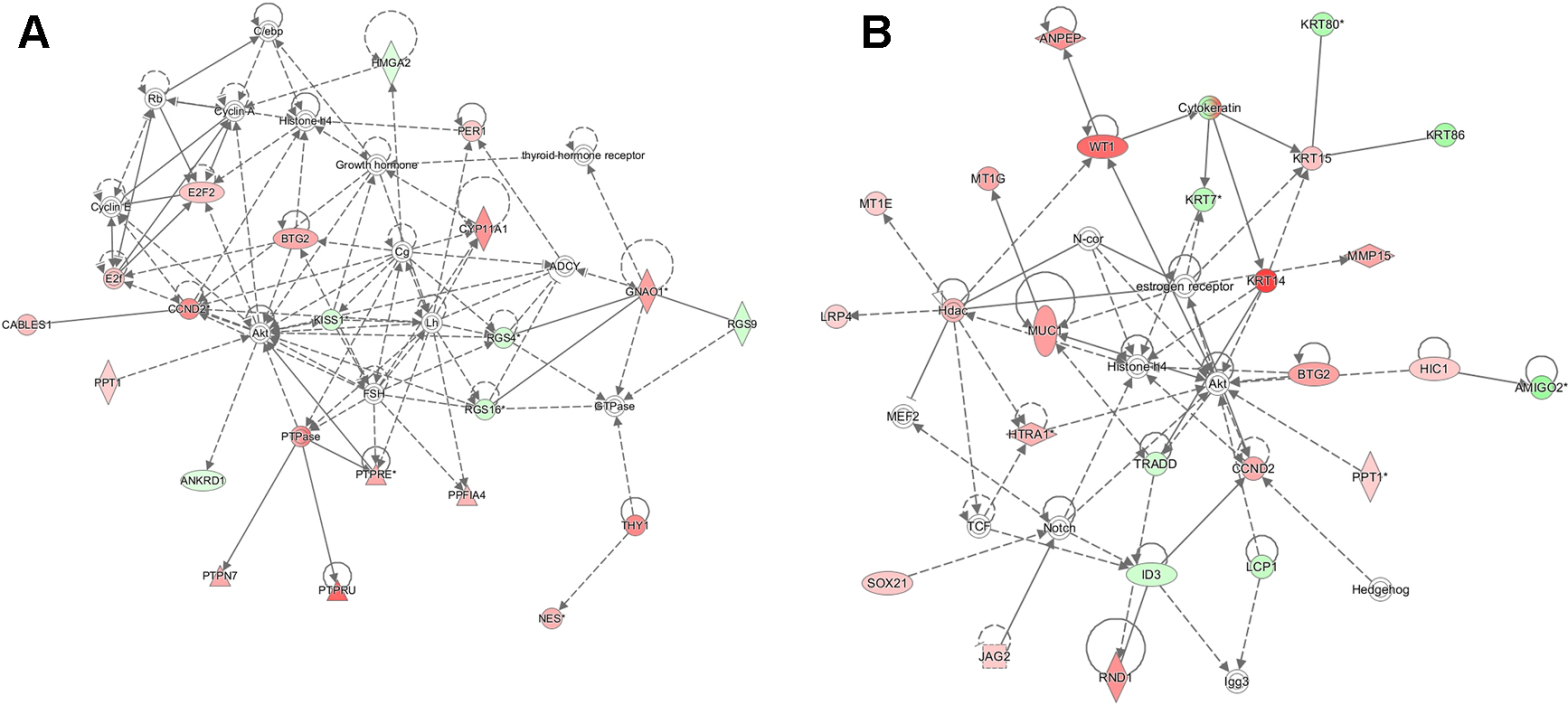
Functional network maps for SIN3 regulated genes Ingenuity pathway analysis (IPA) was utilized to generate functional network maps for the top altered genes following knockdown of SIN3A (**A**) and SIN3B (**B**). Red indicates genes that are up-regulated, green indicates genes that are down-regulated, and white indicates genes that are incorporated into the network through relationships with other genes. The intensity of color indicates the degree of up- or down-regulation. Shapes indicate the type of molecule: horizontal ovals are transcription regulators, vertical ovals are transmembrane receptors, diamonds are enzymes, triangles are phosphatases, double circles are part of complexes or groups, dotted squares are growth factors, and circles are listed as other.

Based on the robust differences observed in the regulation of metastatic potential *in vitro* and *in vivo*, we examined potential differences in the target genes for SIN3A and SIN3B. Total RNA from 2 biological replicates of each cell line (MDA-MB-231 with shSIN3A or shSIN3B) was analyzed by next generation sequencing. Genes that were changed at least 2-fold with *p* < 0.05 compared to control in either of the replicates were identified: 108 were unique for SIN3A knockdown, 125 were unique for SIN3B knockdown, and 39 were common for both SIN3A and SIN3B knockdown (Supplementary Table S2). Using Ingenuity Pathway Analysis (IPA), the top networks that were altered from these gene lists were cell death/survival, cancer, and tumor morphology for SIN3A knockdown, and cancer, cell death/survival, and organismal injury and abnormalities for SIN3B knockdown (Table 1). Functional gene networks were generated using IPA to visualize differences with the connectivity from these gene lists (Figure 4). Although a few genes within the two networks were similar, the overall networks regulated by SIN3A and SIN3B appeared to be quite different. The gene lists were then restricted to those that were altered in both biological replicates (Table 2). The top three most significant diseases and functions for genes in these restricted lists were identified with IPA (Table 3). To further assess the role of SIN3A and SIN3B gene targets, canonical network pathways were identified to determine differences in pathways regulated by SIN3A and SIN3B (Supplementary Figure S3). Together, these results demonstrate differential gene regulation by SIN3A and SIN3B that may help to explain reasons for the functional differences in breast cancer.

**Table 1:** IPA top networks

Top Networks for SIN3A		
	**Associated Network Functions**	**Score**
1	Cell Death and Survival, Cancer, Tumor Morphology	38
2	Liver Hyperbilirubinemia, Metabolic Disease, Developmental Disorder	33
3	Developmental Disorder, Hereditary Disorder, Neurological Disease	28
4	Gastrointestinal Disease, Infectious Disease, Organismal Functions	21
5	Developmental Disorder, Hereditary Disorder, Neurological Disease	19
6	Cell-To-Cell Signaling and Interaction, Carbohydrate Metabolism, Molecular Transport	19
7	Cellular Development, Nervous System Development and Function, Gene Expression	19
8	Cellular Movement, Hematological System Development and Function, Humoral Immune Response	17
9	Connective Tissue Disorders, Immunological Disease, Inflammatory Disease	13
10	Endocrine System Development and Function, Small Molecule Biochemistry, Drug Metabolism	11
11	Connective Tissue Disorders, Developmental Disorder, Endocrine System Disorders	2
12	Cellular Development, Inflammatory Disease, Inflammatory Response	2

Top Networks for SIN3B		
	**Associated Network Functions**	**Score**
1	Cancer, Cell Death and Survival, Organismal Injury and Abnormalities	47
2	Renal and Urological System Development and Function, Cellular Movement, Inflammatory Disease	39
3	Cell Morphology, Cellular Assembly and Organization, Cellular Function and Maintenance	27
4	Reproductive System Development and Function, Cancer, Gastrointestinal Disease	25
5	Cell Death and Survival, Nervous System Development and Function, Cellular Development	25
6	Nucleic Acid Metabolism, Small Molecule Biochemistry, Connective Tissue Development and Function	23
7	Cellular Movement, Embryonic Development, Organismal Development	21
8	Molecular Transport, Skeletal and Muscular System Development and Function, Endocrine System Disorders	16
9	Cell-To-Cell Signaling and Interaction, Molecular Transport, Connective Tissue Development and Function	16
10	Nervous System Development and Function, Neurological Disease, Organismal Injury and Abnormalities	7
11	Cancer, Cell-To-Cell Signaling and Interaction, Immunological Disease	2
12	Connective Tissue Development and Function, Connective Tissue Disorders, Dermatological Diseases and Conditions	2
13	Cancer, Hematological Disease, Immunological Disease	2
14	Cancer, Gastrointestinal Disease, Organismal Injury and Abnormalities	2

**Table 2:** Up- and down-regulated genes after SIN3 knockdown

SIN3A knockdown			
**Gene ID**	**Direction**	**Fold Change**	**Biological Process/Disease Function**
DIO2	Upregulated	5.20	selenium binding
ACOT4	Upregulated	4.14	fatty acid biosynthesis
OASL	Upregulated	3.95	interferon signaling
KRT81	Upregulated	3.11	formation of intermediate filaments
IL24	Upregulated	3.06	migration of cells
S100A2	Upregulated	2.73	migration of cells
OTUB2	Upregulated	2.54	deubiquitination
SHISA2	Upregulated	2.51	FGF and WNT signaling
CLMP	Upregulated	2.26	cell-cell adhesion
NYNRIN	Upregulated	2.85	nucleic acid binding
LINC00460	Upregulated	2.32	lncRNA class association
FOS	Upregulated	2.14	migration of cells
PTPRE	Upregulated	2.81	cell death
EMP2	Downregulated	−2.56	cell death
DZIP1	Downregulated	−2.61	development of connective tissue
RGS4	Downregulated	−3.40	migration of cells
RGS16	Downregulated	−3.79	cell death
PTGFR	Downregulated	−4.31	cell death
KISS1	Downregulated	−3.49	migration of cells
KIRREL3	Downregulated	−9.87	migration of cells

SIN3B knockdown			
**Gene ID**	**Direction**	**Fold Change**	**Biological Process/Disease Function**
IL32	Upregulated	4.91	apoptosis
WNT11	Upregulated	5.89	apoptosis
RBPMS2	Upregulated	2.75	nucleotide binding
TUB	Upregulated	3.56	apoptosis
CDA	Upregulated	3.51	cell death
Gene ID	Direction	Fold Change	Biological Process/Disease Function
IL32	Upregulated	4.91	apoptosis
WNT11	Upregulated	5.89	apoptosis
RBPMS2	Upregulated	2.75	nucleotide binding
TUB	Upregulated	3.56	apoptosis
CDA	Upregulated	3.51	cell death
SPINT1	Upregulated	2.84	apoptosis
MYPN	Upregulated	2.72	neoplasia of epithelial tissue
MAPT	Upregulated	2.65	apoptosis
HTRA1	Upregulated	2.74	apoptosis
B4GALNT4	Upregulated	3.98	neoplasia of epithelial tissue
KCNJ11	Upregulated	2.79	apoptosis
STAT5A	Upregulated	2.72	cell movement
PPM1J	Upregulated	2.96	protein serine/threonine phosphatase activity
GALNT16	Upregulated	2.27	neoplasia of epithelial tissue
DGAT2	Upregulated	2.63	neoplasia of epithelial tissue
FAM86C1	Downregulated	−2.09	neoplasia of epithelial tissue
TINAGL1	Downregulated	−2.09	vascular disease
KRT80	Downregulated	−3.02	neoplasia of epithelial tissue

**Table 3:** IPA top disease and function networks

SIN3A knockdown		
**Consistency Score**	**Diseases and functions**	Target molecules in dataset*
61	angiogenesis, branching of cells, cell death of cancer cells, cell survival, development of body trunk, growth of neurites, growth of tumor, outgrowth of cells, S phase, sarcoma, vascular disease	ABCC3, ANPEP, ATF3, BTG2, CASP1, CCND2, CDH11, COL1A1, CXCL12, CYP11A1, CYP1A1, DCN, E2F2, FOS, GNAO1, GREM1, HBA1/HBA2, IL18, KITLG, MMP1, MMP2, NES, NR4A1, PDE4B, POSTN, PPFIA4, PTPRE, RGS4, RRAD, S1PR1, SLC16A1, SLCO1B3, TINAGL1
35	angiogenesis, cell death of cancer cells, colony formation of cells, development of body trunk, growth of neurites, growth of tumor, S phase, sarcoma, synthesis of hormone, vascular disease	ANPEP, ATF3, BTG2, CASP1, CCND2, CDH11, COL1A1, CXCL12, CYP11A1, CYP1A1, DCN, DIO2, E2F2, FOS, GNAO1, GREM1, HBA1/HBA2, IL18, KITLG, MMP1, MMP2, NES, NR4A1, PDE4B, PTPRE, RGS4, RRAD, S1PR1, SLC16A1, THY1, TINAGL1
33	angiogenesis, branching of cells, cell death of cancer cells, cell survival, colony formation of cells, connective or soft tissue tumor, development of body trunk, growth of neurites, growth of tumor, S phase, vascular disease	ABCC3, ANPEP, ATF3, BTG2, CCND2, CDH11, COL1A1, CXCL12, CYP11A1, CYP1A1, E2F2, FOS, GNAO1, GREM1, HBA1/HBA2, IL18, KITLG, MMP1, MMP2, NES, NR4A1, PDE4B, POSTN, PPFIA4, PTPN7, PTPRE, RGS4, RRAD, S1PR1, SLC16A1, SLCO1B3, THY1

SIN3B knockdown		
**Consistency Score**	**Diseases and functions**	Target molecules in dataset*
22	cell movement of endothelial cells, cell viability, development of epithelial tissue, differentiation of epithelial tissue, interphase, mass of heart, vascularization of body region, vascularization of lesion	ANPEP, ATF3, BTG2, CCND2, CSF3, DCN, GADD45A, GREM1, HDAC5, HTRA1, ID3, KRT14, MAPT, MMP2, NES, NPR1, NR4A1, PTGS2, RASGRF1, RRAD, S100A4, SLC16A1, SLC2A4, SOCS2, TRADD, TSC22D1, WNT10B, WT1
15	apoptosis of breast cancer cell lines, cell movement of endothelial cells, development of epithelial tissue, interphase, organismal death, quantity of phagocytes, vasculogenesis	ANPEP, ATF3, BIRC3, CCND2, CD68, CSF3, EGR2, GADD45A, HDAC5, KRT14, MMP2, MUC1, NES, NR4A1, PTGS2, RASGRF1, S100A4, SLC2A4, TXNIP, WNT10B, WT1
15	development of epithelial tissue, vascularization of body region, vascularization of lesion	ANPEP, CSF3, ID3, MMP2, NR4A1, PTGS2, S100A4, STAT5A, TIE1, WNT10B, WT1

* Red indicates up-regulation and green indicates down-regulation of target genes upon knockdown of SIN3A or SIN3B.

### SIN3 paralog expression correlates with relapse-free survival of patients with triple negative breast cancer

**Figure 5:**
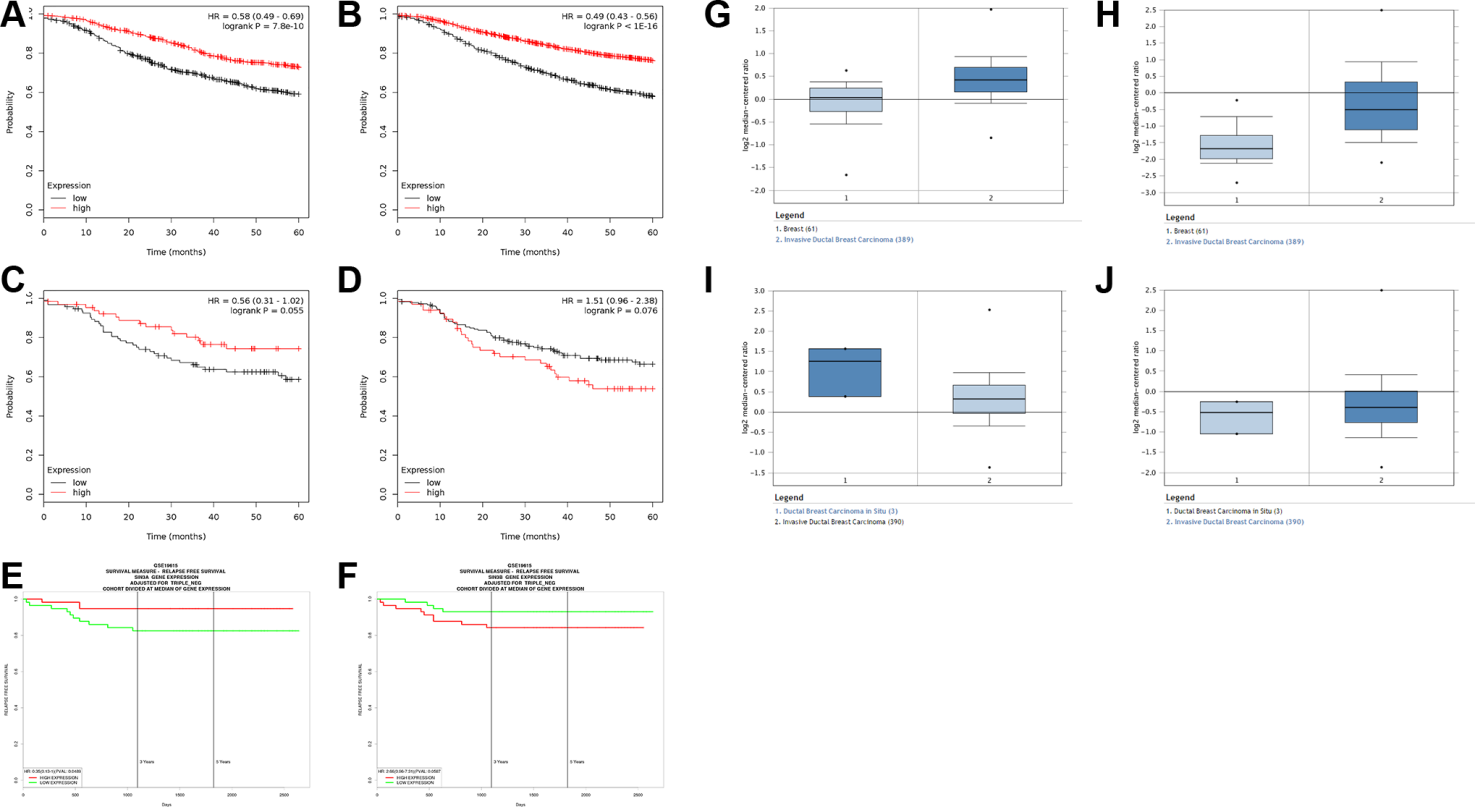
Correlation of SIN3 paralog expression in patients with breast cancer (**A**, **B**) Five-year relapse-free survival rates for all breast cancers with high and low expression of SIN3A (A) and SIN3B (B) generated with the public database kmplot.com (hazard ratios = 0.58 and 0.49; logrank *P* = 7.8e-10 and < 1e-16 respectively for *SIN3A* and *SIN3B*). (**C**–**F**) Five-year relapse-free survival rates specifically in patients with triple-negative breast cancer with high and low expression of *SIN3A* (C,E) and *SIN3B* (D,F) using kmplot. com (C,D) and PROGgenV2 (E,F). High expression of *SIN3A* and *low* expression of *SIN3B* correlated with a longer relapse-free survival specifically in triple negative breast cancers (hazard ratios = 0.56 and 1.51; logrank *P* = 0.055 and 0.076 respectively for *SIN3A* and *SIN3B* using kmplot.com in C,D and hazard ratios = 0.35 and 2.66; logrank *P* = 0.0489 and 0.0587 respectively for *SIN3A* and *SIN3B* using PROGgeneV2 in E,F). Oncomine analysis of normal breast samples compared to invasive ductal carcinoma (IDC) shows increases in the expression of *SIN3A* (**G**) and *SIN3B* (**H**) (*p* = 4.11E-8 and *p* = 5.56E-29, respectively for *SIN3A* and *SIN3B*). However, a trend is noted for decreased *SIN3A* expression (**I**) *p* = 0.084) and increased *SIN3B* expression (**J**) *p* = 0.205) in patients with IBC compared to ductal carcinoma *in situ* (DCIS).

It is not yet clear whether SIN3 paralog expression correlates with survival of patients with breast cancer. To address this, we utilized a Kaplan Meier plotter database (www.kmplot.com) [22]. We compared *SIN3A* and *SIN3B* gene expression with relapse-free survival of patients with breast cancer. High expression of either *SIN3A* or *SIN3B* correlated with longer relapse-free survival in all breast cancers (hazard ratios = 0.58 and 0.49; logrank *P* = 7.8e-10 and < 1e-16 respectively for *SIN3A* and *SIN3B*) (Figure 5A, 5B). However, high expression of *SIN3A* and *low* expression of *SIN3B* correlated with a longer relapse-free survival specifically in triple negative breast cancers (hazard ratios = 0.56 and 1.51; logrank *P* = 0.055 and 0.076 respectively for *SIN3A* and *SIN3B*) (Figure 5C, 5D). We also utilized the PROGgeneV2 database (watson.compbio.iupui.edu/chirayu/proggene/database/index.php) [23, 24] with similar results of high expression of *SIN3A* and low expression of *SIN3B* correlated with longer relapse-free survival specifically in triple negative breast cancers (hazard ratios = 0.35 and 2.66; logrank *P* = 0.0489 and 0.0587 respectively for *SIN3A* and *SIN3B*) (Figure 5E, 5F). Furthermore, we analyzed the *SIN3A* and *SIN3B* unique targets and found that for several of these genes the expression level corresponded to the expected relapse-free survival (Supplementary Figure S4). Additionally, we compared invasive breast carcinoma (IBC) to ductal breast carcinoma *in situ* (DCIS) from patients with triple negative breast cancer using Oncomine (TCGA breast database). *SIN3A* and *SIN3B* expression is significantly increased (*p* = 4.11E-8 and *p* = 5.56E-29, respectively) compared to their normal counterparts (Figure 5G, 5H). However, we noted a trend with decreased *SIN3A* expression (*p* = 0.084) and increased *SIN3B* expression (*p* = 0.205) in patients with IBC compared to DCIS (Figure 5I, 5J). Although statistical significance was not reached, the data is consistent with survival data analyzed from kmplot.com and PROGgeneV2 for triple negative breast cancers and correlates well with our *in vitro* and *in vivo* findings.

## DISCUSSION

SIN3 has been shown to be a key scaffold central to the recruitment of multiprotein complexes that epigenetically regulate gene transcription [11]. As such, the expression of SIN3 paralogs has been implicated in both normal development and cancer progression. However, distinct functions for these paralogs have not yet been characterized. In this study, we demonstrate differential roles of SIN3 paralogs in breast cancer progression and metastatic potential. Our data supports SIN3A as a suppressor of breast cancer progression and metastasis, whereas SIN3B may be a promoter. Knockdown of SIN3A caused an increase in the number of invaded cells, the presence of invasive colonies in 3D, and increased metastatic potential of breast cancer cells. SIN3B knockdown decreased invasiveness, promoted a more epithelial-like morphology in 3D, and decreased metastatic potential. In addition to the phenotypic differences, we also found differences in unique target genes for these paralogs. Moreover, we identified correlations of high *SIN3A* and low *SIN3B* mRNA expression with relapse-free survival specifically in triple negative breast cancers.

The differential roles of SIN3A and SIN3B in breast cancer progression is not completely unexpected considering that knockout mice for either *Sin3A* or *Sin3B* is embryonic lethal at different stages of development. Knockout of *Sin3A* leads to embryonic lethality at embryonic day 6.5, whereas knockout of *Sin3B* is embryonic lethal by postnatal day 1 [12–14]. A direct comparison of *Sin3A* and *Sin3B* was performed by crossing mice expressing floxed alleles of *Sin3A* and *Sin3B* with Cre expressing mice in the myoblast compartment (*Myf5-Cre*) or differentiated skeletal muscle cells (*MCK-Cre*) [15]. Gross defects in sarcomere structure were noted with *Sin3A* conditional knockout in myotubes, but not with *Sin3B* knockout. The defects were significantly enhanced with simultaneous knockout of both *Sin3A* and *Sin3B*. Several distinct phenotypes have also been noted using other conditional knockout models [14][12][13]. Although mechanisms for these distinct phenotypes in normal development have not yet been fully characterized, it is clear that SIN3A and SIN3B have distinct functions.

Lowered expression levels of SIN3A and SIN3B have also been associated with the progression of many cancers [20]. In that study, microarray data from Oncomine was analyzed and showed that *SIN3A* mRNA was significantly reduced in lung, renal, liver, and gastric tumors and lymphoma compared to normal tissue. It was also reduced in breast tumors although statistical significance was not demonstrated. Additional patient samples from lung tumors (*n* = 12) revealed significantly lowered expression of both *SIN3A* and *SIN3B* mRNA compared to non-diseased lung tissue. Suzuki *et al.* showed that *SIN3A* mRNA is more frequently decreased in non-small cell lung cancer patient samples that further supports low expression of *SIN3A* is associated with a more aggressive tumor progression [25]. Consistent with this, knockdown of *SIN3A* resulted in increased invasion in *Drosophila* and increased migration in A549 human lung adenocarcinoma cells [20]. However, knockdown of *SIN3A* in estrogen receptor positive (ER+) breast cancer cell lines resulted in increased apoptosis and attenuation of cell growth that was not identified in ER negative breast cancer cell lines [26]. This suggests that expression and/or functional differences of SIN3 complexes exist between molecular subtypes of breast cancer. Our data shows that high expression of either *SIN3A* or *SIN3B* correlates with longer relapse-free survival of patients with breast cancer when samples were not stratified. Interestingly, when analyzing triple negative breast cancers specifically, we noted that high *SIN3A* or low *SIN3B* mRNA expression correlated with longer relapse-free survival. Although significance was not attained in those analyses, the trend supports our *in vitro* and *in vivo* data for which we used triple negative breast cancer cell lines. In support of this, data analyzed from Oncomine revealed a trend towards decreased expression of *SIN3A* and increased expression of *SIN3B* in patients with triple negative invasive ductal carcinoma compared to ductal carcinoma *in situ*. Overall, this data supports that *SIN3A* and *SIN3B* expression levels in cancer patients will depend on molecular subtype; and in triple negative breast cancer, lowered expression of *SIN3A* and higher expression of *SIN3B* may be associated with more aggressive disease progression.

We identified several genes that are uniquely altered with lowered levels of SIN3A or SIN3B. Although we have not validated these gene sets by analyzing the expression of individual genes, the data support our hypothesis that SIN3A and SIN3B have differential functions in breast cancer progression. It is also interesting to note that expression of several SIN3A and SIN3B target genes in our data sets are consistent with the relapse-free survival identified for SIN3A and SIN3B for patients with triple negative breast cancer. Many of the genes in our list are of interest for follow-up study because of their known functions related to cancer. For example, high expression of RGS4, which was downregulated in our SIN3A knockdown gene list, has been associated with inhibition of migration, invasion and delayed tumor growth of MDA-MB-231 cells [27]. KISS1, a validated metastasis suppressor, [28] was also downregulated in our SIN3A knockdown gene list. HTRA1 was upregulated on the SIN3B knockdown gene list and high HTRA1 expression is associated with overall and disease-free survival in patients with breast cancer [29]. STAT5A was also increased in the SIN3B knockdown gene list. Low levels of STAT5A are associated with breast cancer progression and poor prognosis of patients with breast cancer [30]. Additionally, Das *et al*. showed that low levels of SIN3A is associated with tumor progression and is associated with upregulation of genes involved in cell migration and invasion [20]. This correlates well with our functional *in vitro* invasion data although the identified genes did not match with those on our lists. It is quite possible this is because the model systems used were different. Future work will be necessary to fully understand specific targets regulated by SIN3A and SIN3B and in what context or molecular subtype of breast cancer.

In summary, our results demonstrate paralog-specific functions for SIN3 in breast cancer progression. Importantly, we found differences in the genes they regulate and possible correlations with patient samples. As many groups are developing therapeutic strategies for metastasis, we provide here a caution for two proteins that have been thought to be very similar with regard to sequence, protein-protein interactions, and function. SIN3 has recently gained interest as a potential drug target [7, 8]. The studies presented here could prove meaningful during the developmental drug discovery pipeline and provide a foundation for moving forward with relevant target identification for metastatic breast cancer.

## MATERIALS AND METHODS

### Cells and cell culture

Triple negative human breast carcinoma cell lines, MDA-MB-231 (231), MDA-MB-435 (435), and MDA-MB-436 (436) were previously described [19, 40]. The origin of the 435 cell line has been questioned in the past because the cells express melanoma-associated genes [31, 32]. However, we believe the overall data is consistent with it being breast carcinoma [33, 34] including the ability to secrete milk lipids [35, 36] and the propensity to metastasize from the mammary fat pad and not from subcutaneous sites [37]. Nonetheless, the origin of 435 does not affect the interpretation of the results presented. The cells were cultured in a 1:1 mixture (v/v) of Dulbecco's modified Eagle's medium (DMEM) and Ham's-F12 medium (DMEM/F12; Invitrogen, Carlsbad, CA) supplemented with 2 mM L-glutamine, 0.02 mM nonessential amino acids and 10% fetal bovine serum (Invitrogen). Cells were maintained without antibiotics nor antimycotics on 100-mm tissue culture dishes (Corning, Corning, NY) at 37°C with 5% CO_2_ in a humidified atmosphere. Cells were routinely passaged using a solution of 2 mM EDTA in Ca^2+^/Mg^2+^-free Dulbecco's phosphate buffered saline (CMF-DPBS; Invitrogen). All cultures were regularly tested and confirmed negative for *Mycoplasma* spp. infection using a cell-based colorimetric assay (PlasmoTest, InvivoGen, San Diego, CA).

### Constructs and transduction

Cells were stably transduced with one of 3 non-overlapping shRNA targeting sequences located in exon 19, 11, and 15–16 of *SIN3A* (sh1, sh2 and sh3 respectively) and exon 4, 20, and 3 of *SIN3B* (sh1, sh2, and sh3 respectively) (see Supplementary Table S1 for specific sequences). Constructs targeting *SIN3A* were inserted into the pSilencer 5.1-U6 retroviral vector (pSilencer; Life Technologies, Grand Island, NY) that contains puromycin resistance. To generate hygromycin resistance for constructs targeting *SIN3B*, the pSilencer 5.1-U6 retroviral vector sequence was mutated to include MluI and BglII restriction enzyme sites to replace puromycin resistance with hygromycin resistance which was digested from the pMCSV hygromycin retroviral vector (Clontech Laboratories, Mountain View, CA) using BglII and MluI restriction enzymes. We also generated a dual knockdown cell line which we termed B_2_A_3_ in which we used sh2 targeting *SIN3B* and sh3 targeting *SIN3A* to knockdown both *SIN3A* and *SIN3B* in the 231 and 435 cell lines. Retroviral vectors were packaged in 293 GPG cells using Lipofectamine 2000 (ThermoFisher Scientific, Grand Island, NY) and supernatants were collected at 24, 48, 72, and 96 hours. Cells were transduced with 1 mL of selected virus and 40 μL of polybrene. Transduced cells were initially selected with puromycin (2 ng/ml) and/or hygromycin (500 μg/μL) and maintained in puromycin (1 ng/μL) or hygryomycin (250 μg/μL) to ensure stable transduction. For routine culture and experiments no antibiotics were added.

### Cell proliferation

Proliferation assays were performed as previously described [19, 38]. Briefly, cells were seeded (500–5000) in 96-well tissue culture plates. Cell proliferation was assessed at days 1, 3, 5, and 7 by measuring fluorescence after addition of AlamarBlue Reagent (Life Technologies, Carlsbad, CA). Fluorescence intensity was quantified at 570/585 nm (excitation/emission) using a Hitachi F-7000 fluorescence spectrophotometer.

### Western blot analysis

Cells were grown on 10 cm tissue culture plates and whole cell lysates were collected with 1X RIPA buffer supplemented with protease inhibitor cocktail (ThermoFisher Scientific). Lysates were separated with Bis-Tris gels (GenScript, Township, NJ and Biorad, Hercules, CA), transferred to PVDF membranes and blocked in 5% non-fat milk in tris-buffered saline with 0.05% tween-20 (TBST) for 1 hour. A SIN3A polyclonal antibody was generated by 21^st^ Century Biochemicals using a synthetic peptide corresponding to amino acids 444–460 of human SIN3A followed by affinity purification. Validation was performed by western blot, immunoprecipitation and mass spectroscopy. The following dilutions were used: SIN3A (1:2000), SIN3B (1:1000, Santa Cruz Biotechnology, Santa Cruz, CA), β-actin (1:10,000, Sigma-Aldrich, St. Louis, MO) or GAPDH (1:2000, Cell Signaling Technology, Danvers, MA). All primary antibodies were incubated overnight at 4°C with shaking. Secondary antibodies (donkey anti-rabbit IgG or sheep anti-mouse; 1:10,000, GE Healthcare Life Sciences, Chalfont, UK) were incubated for 1 hour at room temperature. Pierce ECL Western Blotting Substrate or Pierce West Dura Extended Duration Substrate (ThermoFisher Scientific) was used to develop blots on chemiluminescence film. Densitometry analysis was performed using ImageJ software (NIH, Bethesda, MD) and relative SIN3A or SIN3B band intensity normalized to β-actin was quantified with respect to the scrambled control (scr).

### Cell invasion

Invasion assays were performed as described previously [39]. Briefly, transwell chambers with 24-well inserts coated with Matrigel (8 μm diameter pores; BD Biosciences, San Jose, CA) were used. Cells were seeded (5 × 10^5^ cells/insert) and invaded cells were stained with 1% crystal violet after 22 hours.

### Three-dimensional growth

To assess 3D growth, 24-well tissue culture plates were coated with 400 μL of reduced growth factor Matrigel (BD Biosciences). Cells were suspended in complete culture medium supplemented with 4% Matrigel and seeded at a density of 2000 cells/well. Every 4 days, the media was replaced with fresh complete culture medium supplemented with 2% Matrigel. Images were captured after incubation at 37°C for 10 days with a Nikon Eclipse TE2000-U microscope.

### Animal models

Metastasis assays were performed as previously described [40]. Briefly, 5 × 10^5^ cells in 0.2 mL HBSS were injected into the lateral tail vein of 5 week old female Nu/J athymic mice (The Jackson Laboratory, Bar Harbor, ME). Lungs were collected after 8 weeks and stained in Bouin's fixative. Metastases per lung were counted and the average number was normalized to control (parental cell line). Animals were maintained under the guidelines of NIH and the University of Alabama at Birmingham Institutional Animal Care and Use Committee. Food and water were provided *ad libitum*.

### Next generation sequencing

Cells were grown on 15 cm tissue culture plates and total RNA was collected with Trizol reagent (Life Technologies, Carlsbad, CA). RNA pellets were resuspended in DEPC-treated water and 200 ng/μL was sequenced at the Heflin Genetics Core facility at the University of Alabama at Birmingham as follows. mRNA-sequencing was performed on the Illumina HiSeq2500 using the latest versions of the sequencing reagents and flow cells providing up to 300 Gb of sequence information per flow cell. The quality of the total RNA was assessed using the Agilent 2100 Bioanalyzer followed by 2 rounds of poly A+ selection and conversion to cDNA. The TruSeq library generation kits were used as per the manufacturer's instructions (Illumina, San Diego, CA). Library construction consisted of random fragmentation of the poly A mRNA, followed by cDNA production using random primers. The ends of the cDNA were repaired, A-tailed and adaptors ligated for indexing (up to 12 different barcodes per lane) during the sequencing runs. The cDNA libraries were quantitated using qPCR in a Roche LightCycler 480 with the Kapa Biosystems kit for library quantitation (Kapa Biosystems, Woburn, MA) prior to cluster generation. Clusters were generated to yield approximately 725K-825K clusters/mm^2^. Cluster density and quality were determined during the run after the first base addition parameters were assessed. Paired end 2 × 50 bp sequencing runs were used to align the cDNA sequences to the reference genome.

TopHat was used to align the raw RNA-Seq fastq reads to the human hg19 genome using the short read aligner Bowtie [41–43]. Cufflinks was used to align the reads from TopHat to assemble transcripts, estimate their abundances and test for differential expression and regulation [43, 44]. The assembled transcripts were merged to a reference annotation using Cuffmerge. Finally, Cuffdiff was used to identify significant changes in transcript expression, splicing and promoter use. Genes with fold change ≥ ±2.0 and *p* < 0.05 were further analyzed using Ingenuity's Pathway Analysis tool.

### Analysis of SIN3 paralog expression in patient samples

The Kaplan Meier Plotter database (kmplot.com) [22] and PROGgeneV2 database (watson.compbio.iupui.edu/chirayu/proggene/database/index.php; GSE19615) [23, 24] were used to investigate correlations of *SIN3A* and *SIN3B* expression with patient relapse-free survival. The databases use gene expression data from public repositories including GEO (affymetrix microarrays), EGA, and TCGA. Patients were split into high and low gene expression groups based on median gene expression. The two patient cohorts were then compared by Kaplan-Meier survival plots and the hazard ratio and logrank *P* value calculated. The Oncomine database (www.oncomine.org) was used to determine correlations between *SIN3A* and *SIN3B* expression with breast cancer progression (breast cancer versus normal breast and invasive breast cancer versus ductal carcinoma *in situ*).

### Statistical analysis

Statistical analyses were performed with SigmaStat software version 3.5. Mann-Whitney rank sum test or Kruskal-Wallis one-way ANOVA using the Student-Newman-Keuls method for multiple comparisons were performed for the invasion and metastases assays. A *p* ≤ 0.05 was considered statistically significant.

## SUPPLEMENTARY MATERIALS




