# Supplementary material for: SIN3A and SIN3B differentially regulate breast cancer metastasis
Source: Oncotarget. 2016 Oct 21;7(48):78713–25. doi: 10.18632/oncotarget.12805 (PMC5340233; doi:10.18632/oncotarget.12805)
Supplement: Supplementary file 1 [file oncotarget-07-78713-s001.pdf]

SIN3A and SIN3B differentially regulate breast cancer metastasis

Supplementary Materials

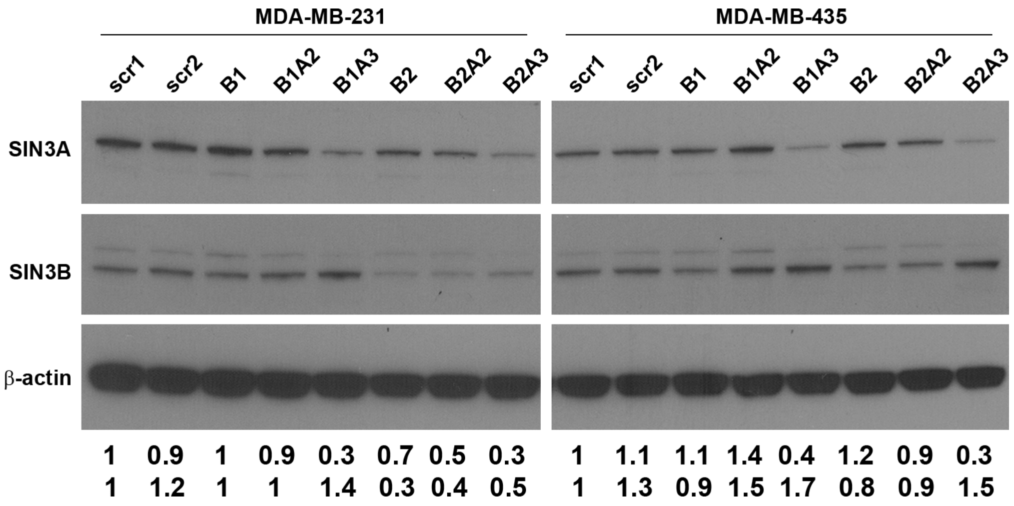

Supplementary Figure S1: Western blot analysis of whole cell lysates from MDA-MB-231 and MDA-MB-435 cells. Knockdown of SIN3A and SIN3B using three different non-overlapping shRNAs: sh1, sh2, and sh3 (sequences and location shown in supplementary table 1). Dual knockdown was achieved using sh1 and sh2 targeting SIN3B and sh2 and sh3 targeting SIN3A. SCR indicates vector controls with scrambled sequence. β-actin was used as loading control. Densitometry is shown below the blots for SIN3A (top row) and SIN3B (bottom row).

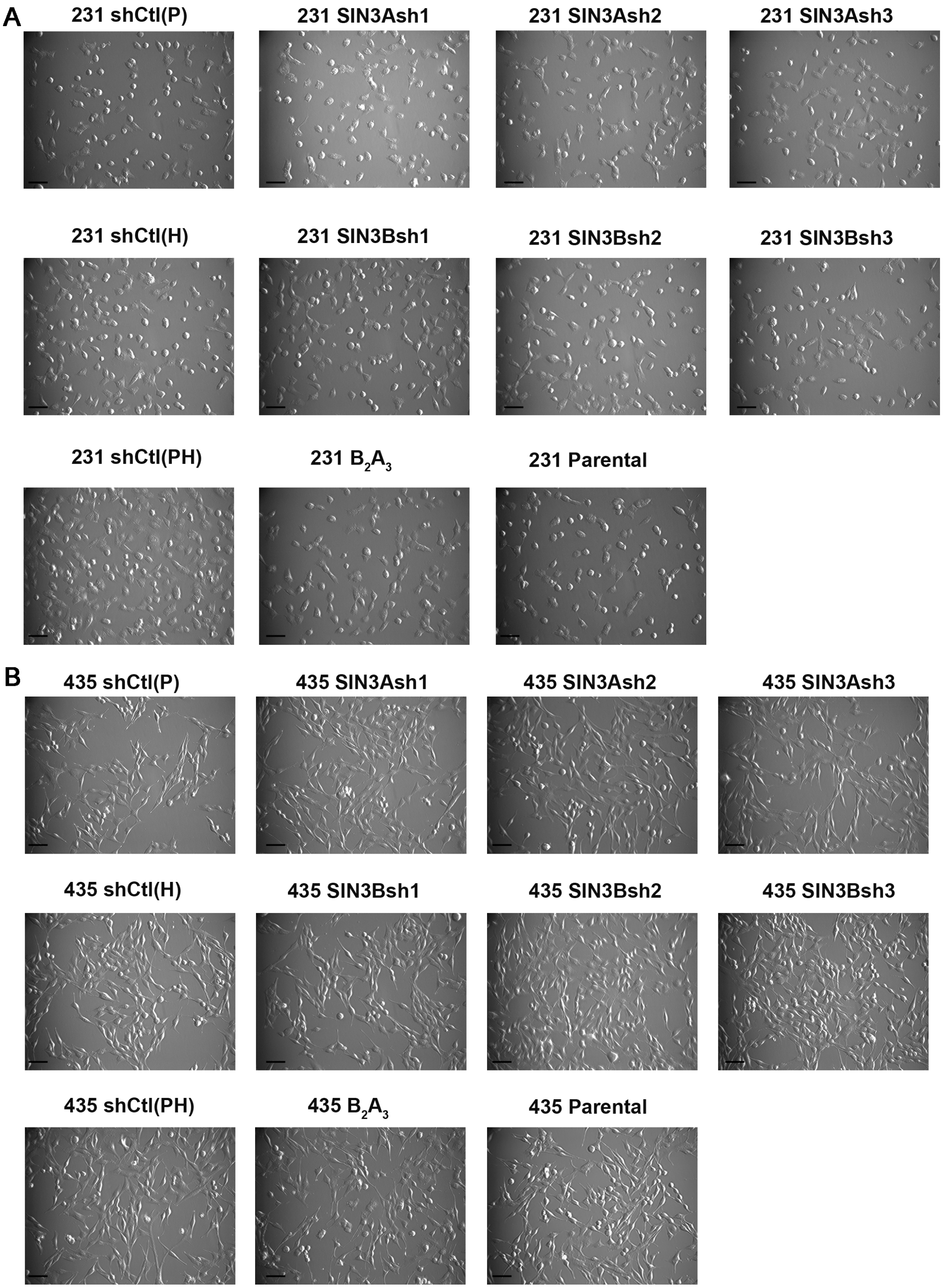

Supplementary Figure S2: Representative brightfield images of MDA-MB-231 (**A**) and MDA-MB-435 (**B**) showing vector control (shCtl(P) is puromycin resistance vector, shCtl(H) is hygromycin resistance vector, and shCtl(PH) is combined puromycin and hygromycin resistant), SIN3A knockdown, SIN3B knockdown, and parental cells grown on 2D tissue culture plates. Scale bar is 50 μm.

A

Canonical pathways regulated by SIN3A knockdown

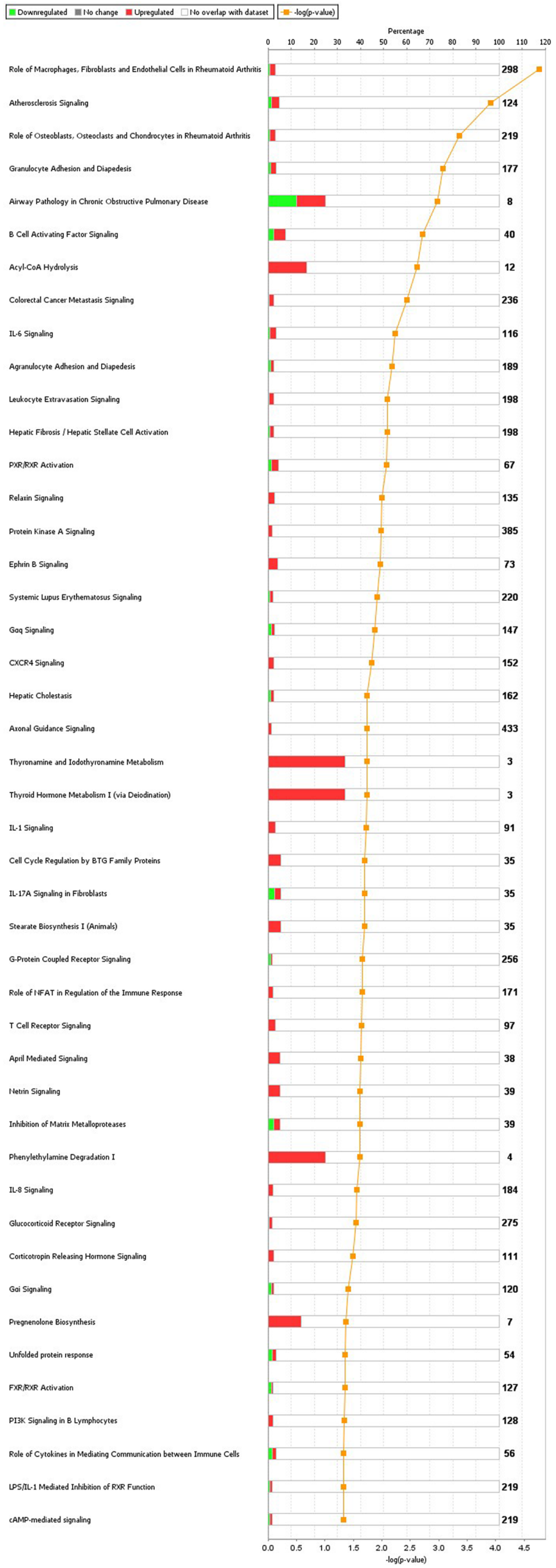

B
Canonical pathways regulated by SIN3B knockdown

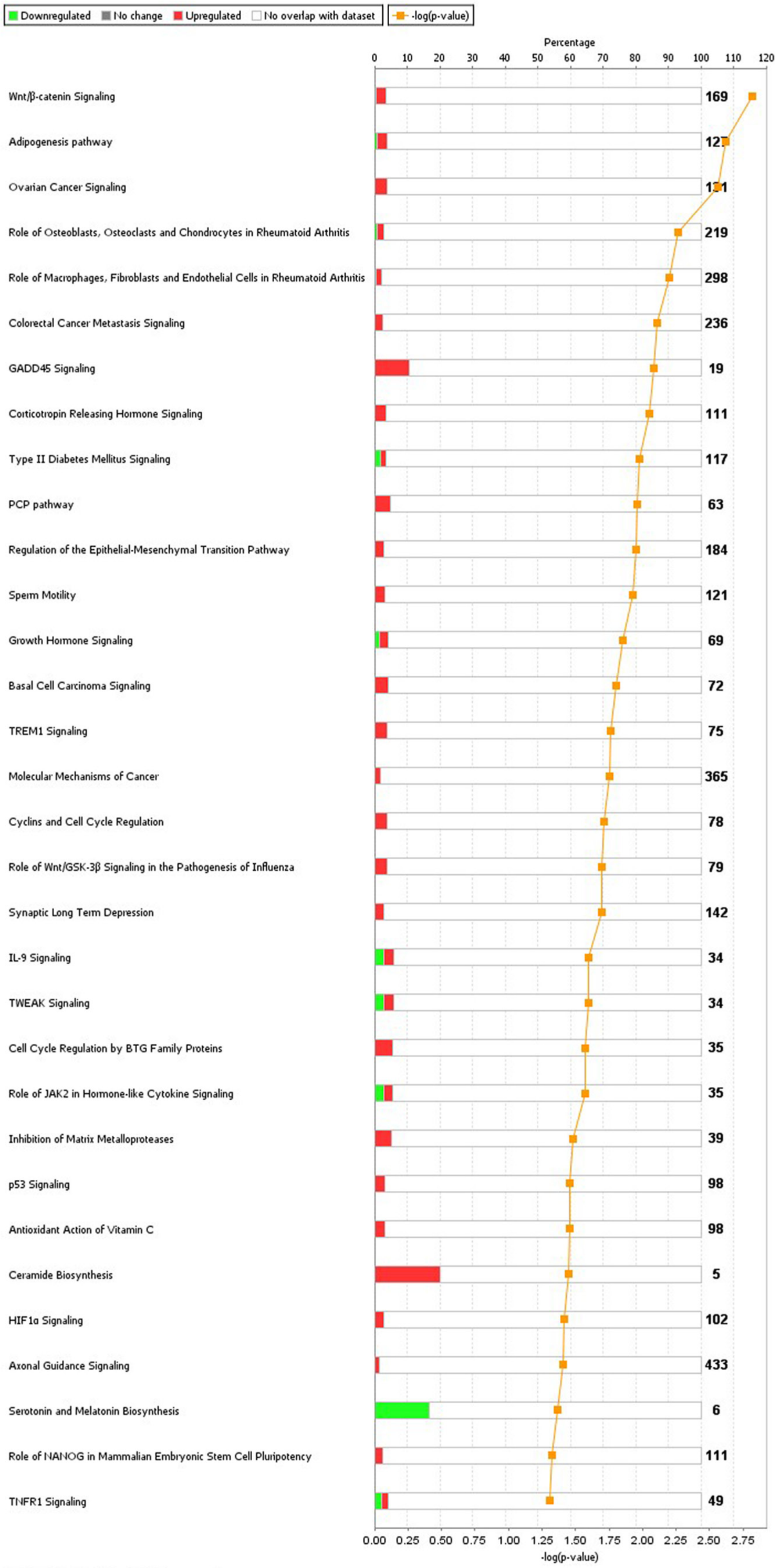

Supplementary Figure S3: Canonical pathways identified through IPA. (**A**) Pathways regulated by SIN3A knockdown and (**B**) pathways regulated by SIN3B knockdown. Total number of molecules identified in each pathway is noted to the right of each bar. Green indicates the percentage of molecules down-regulated and red indicates the percentage of molecules up-regulated by SIN3A or SIN3B knockdown. Orange squares represent [–log(p value)] for each pathway. Fisher's Exact Test p-value was used to test significance and canonical pathways with $p < 0.05$ are shown.

Upregulated with SIN3A knockdown

OTUB2

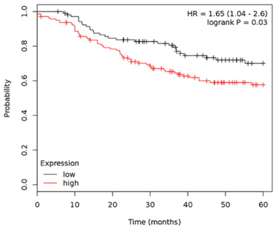

S100A2

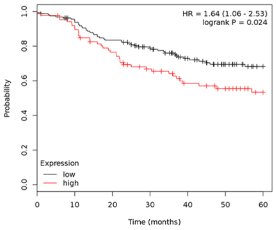

Upregulated with SIN3B knockdown

GALNT16

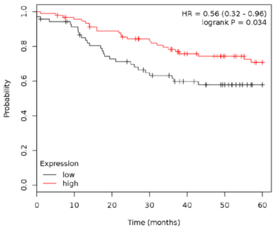

IL32

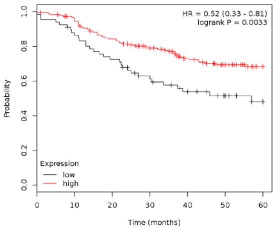

MYPN

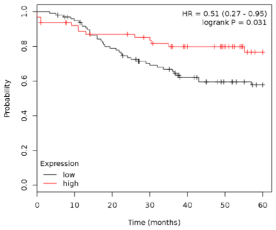

STAT5A

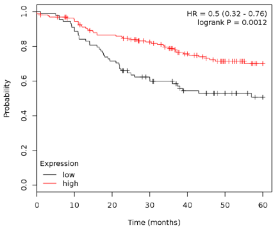

WNT11

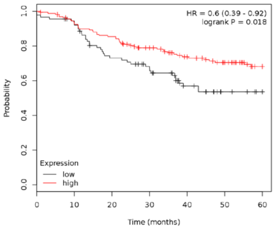

Downregulated with SIN3B knockdown

KRT80

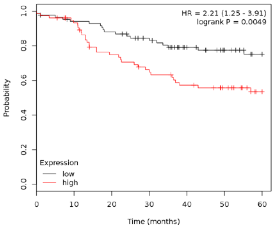

Supplementary Figure S4: Kaplan Meier relapse-free survival plots for patients with triple negative breast cancer generated from kmplot.com for expression of relevant genes identified in our RNAseq analysis that significantly correlated with our *in vitro* and *in vivo* data.

Supplementary Table S1: Sequences for shRNA constructs

SIN3A shRNA	shRNA sequence	Exons targeted	cDNA positions
SIN3Ash1	5′-CGTGAACATCTAGCACAGAAA-3′	Exon 19	3661–3681
SIN3Ash2	5′-CCCTGAGTTGTTTAATTGGTT-3′	Exon 11	1848–1868
SIN3Ash3	5′-AGCTACGTCTCAAAGAACCTAT-3′	Exon 15-16	3147–3167
**SIN3B shRNA**	**shRNA sequence**		
SIN3Bsh1	5′-CAAGCAGCAGGTGCCGTATAA-3′	Exon 4	434–454
SIN3Bsh2	5′-CGCACAGATATTTATTCCTTT-3′	Exon 20	4311–4331
SIN3Bsh3	5′-CCTCGGATATAGAATAGACAT-3′	Exon 3	329–349

Supplementary Table S2: Unique gene expression changes following SIN3A knockdown. See Supplementary_Table_S2.
